# University students who were men who had sex with men (MSM) in Sichuan, China had a higher prevalence of insomnia and probable depression than their non-MSM counterparts: mediation via emotional dysregulations

**DOI:** 10.1186/s12888-024-06192-2

**Published:** 2024-11-12

**Authors:** Yanqiu Yu, Joyce Hoi-Yuk Ng, Zixin Wang, Xiaobing Tian, Joseph T. F. Lau

**Affiliations:** 1 Department of Preventive Medicine and Health Education, School of Public Health, Fudan University, Shanghai, China; 2 Center for Health Behaviours Research, Jockey Club School of Public Health and Primary Care, the Chinese University of Hong Kong, Hong Kong, China; 3 Department of Epidemiology and Biostatistics, School of Public Health, North Sichuan Medical College, Nanchong, China; 4 Zhejiang Provincial Clinical Research Center for Mental Disorders, The Affiliated Wenzhou Kangning Hospital, Wenzhou Medical University, Wenzhou, China; 5 Public Mental Health Center, School of Mental Health, Wenzhou Medical University, Wenzhou, China

**Keywords:** Men who have sex with men, Depression, Insomnia, Emotional regulation, University students, China

## Abstract

**Background:**

Men who have sex with men studying in universities (MSM-US) frequently face multiple sexual minority stressors that potentially lead to maladaptive emotional regulations and mental problems. This study compared the prevalence of depression/insomnia between MSM-US and non-MSM male university students (NUS) and hypothesized that the potential differences would be mediated via emotional dysregulation styles (rumination and catastrophizing).

**Methods:**

The study design was a cross-sectional study. NUS were recruited from a university-based survey using cluster sampling in three universities in China from June to October 2018, while MSM-US from the same university-based survey and the other community-based survey using convenience sampling. The effective samples size was 2,531 (292 MSM-US and 2,239 NUS). Structural equation modeling (SEM) was performed.

**Results:**

MSM-US had significantly higher prevalence of both probable depression (55.1% versus 35.7%; OR = 4.85, 95% CI: 3.38–6.94) and moderate-to-severe clinical insomnia (17.3% versus 4.1%; OR = 2.21, 95% CI: 1.73–2.83) than NUS. MSM-US were also more likely than NUS to use emotional dysregulation styles (rumination/catastrophizing), which were correlated with probable depression/insomnia (*r* = 0.17 to 0.31). In the SEM, the differences in depression/insomnia between MSM-US and NUS were partially mediated by the latent variable of emotional dysregulation (rumination and catastrophizing), with effect sizes of 55.0% for probable depression and 33.6% for insomnia, respectively.

**Conclusions:**

Depression and insomnia were prevalent among male university students in Sichuan, China. Furthermore, MSM-US were at increased risk than NUS for both mental problems; emotional dysregulation partially explained such differences. Future studies are warranted to confirm the findings, develop tailored interventions to address general and MSM-specific stressors and reduce rumination and catastrophizing, and examine whether similar patterns exist in other sexual minority groups.

## Introduction

According to the Minority Stress Theory, sexual minority groups (e.g., men who have sex with men [MSM]) encounter numerous hostile stressors, such as prejudice and discrimination, which trigger maladaptive stress responses adversely affecting physical and mental health [1]. In particular, MSM are vulnerable to depression which is the leading cause of disability and serious negative outcomes (e.g., suicidal ideation and substance use) and contributes greatly to the global disease burden [2]. The prevalence of depression among MSM was disproportionately high; a meta-analysis reported that the pooled prevalence of depression among MSM was 35% in the globe [3]; it was 40.0% among Chinese MSM [4]. In addition, university students are a high-risk group for depression due to various academic and psychosocial pressures [5]; the global prevalence of depression in this population has nearly doubled over the past decade [6], raising an important public health concern. Given the above, university students who are men who have sex with men (MSM-US) are at cumulatively high risk of depression due to multiple stressors related to both college life and MSM status [7]; the prevalence of depression in Chinese MSM-US ranged from 27.2 to 36.1% [4].

Insomnia is another important public health concern that is associated with increased morbidity and mortality rates, impaired cognitive abilities, poor physical health (e.g., cardiovascular diseases), and mental distress [8–10]. Insomnia is prevalent among MSM; 34.6% of MSM participants in a U.K. survey reported poor sleep quality [9]. Insomnia is also a common mental problem among general university students, possibly due to academic stress, poor sleep habits, and adjustment problems [11]. The pooled global prevalence of insomnia was 18.5% among university students [12]; it ranged from 16.4 to 27.5% in China [13]. Similar to depression, MSM-US is at an accumulative risk of insomnia. To our best knowledge, no studies have investigated the prevalence and factors of insomnia among MSM-US.

Notably, insomnia and depression are two strongly correlated, yet distinct mental problems in terms of core symptoms, etiology, and treatment approaches [14, 15]. Given the above high prevalence of depression and insomnia in both MSM and university students, investigating depression and insomnia simultaneously could enhance the understanding of their shared determinants and mechanisms, which would facilitate the devise of more effective and integrated intervention programs. Furthermore, MSM worldwide had a greater risk of HIV transmission [16], and the prevalence of HIV is rapidly increasing in MSM-US [17]. It is warranted to investigate whether mental problems (depression and insomnia) would be more prevalent among MSM-US than their non-MSM counterparts, as HIV and mental problems were closely related and form a syndemic, which is defined as the coexistence of two major health problems affecting each other adversely [18]. As both depression and insomnia were strongly associated with maladaptive HIV-related behaviors (e.g., condomless anal sex) among MSM [19–23], the reduction of depression and insomnia could potentially contribute to the control of the emerging HIV epidemic among MSM-US. In the literature, although many studies investigated mental problems among MSM, only four studies made direct comparisons in the prevalence of depression between MSM and non-MSM in a single study and these studies found a higher prevalence of depression in MSM than non-MSM [24–27]; no studies compared the prevalence of insomnia between MSM and non-MSM in a single study.

Furthermore, to our knowledge, no study was found to identify mediators explaining the differences in mental problems between MSM and non-MSM. The present study contended that the difference in depression/insomnia between MSM-US and non-MSM male university students (NUS) would be mediated by two emotional dysregulation approaches of rumination and catastrophizing. Emotional dysregulation refers to the inability to manage one’s emotional state/experiences. Specifically, rumination refers to over-focusing on passive and perseverative thoughts and feelings related to a negative event [28], while catastrophizing means exaggerating the negative consequences of an event or a decision [29]. The proposed mediations were supported by theoretical and empirical evidence. Theoretically, Hatzenbuehler’s psychological mediation framework proposes that stigma-related stress of sexual minority groups would induce emotional dysregulation and other cognitive problems that would, in turn, increase the risk of psychopathology, depression and insomnia in this case [30]. Empirically, sexual minority groups were more likely than mainstream people to adopt emotional dysregulation such as rumination and catastrophizing approaches [31]; a longitudinal study reported that sexual minority adolescents in the U.S. exhibited greater emotional dysregulation of rumination than their heterosexual peers [32]. Furthermore, the emotion-based theories of depression propose that emotional dysregulation would disturb interpersonal relationships and behavioral processes in daily functioning, and would then increase one’s vulnerability to mental problems (depression and insomnia) [33]. Empirical evidence showed that emotional dysregulation of rumination and catastrophizing were positively associated with both depression [34, 35] and insomnia [36, 37]. To our knowledge, no study tested the mediations explaining the differences in depression/insomnia between MSM-US and NUS via rumination and catastrophizing. This study hence aimed to fill this knowledge gap.

**Fig. 1:**
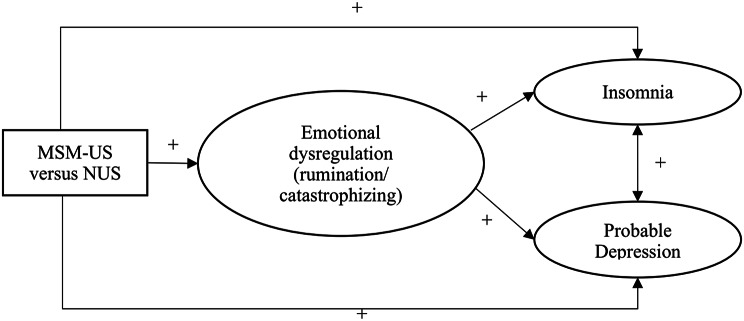
The conceptual mediation model (MSM-US = Men who have sex with men who are university students; NUS = Non-MSM male university students. Background factors were adjusted including age, place of residence, study major, year of study, and average monthly expenses)

Given the aforementioned background, this study compared the prevalence of probable depression and insomnia between MSM-US and NUS in Sichuan, China. The associations between two types of emotional dysregulation (i.e., rumination and catastrophizing) and both probable depression and insomnia were tested. In addition, this study tested the mediation effect of rumination/catastrophizing on the differences in probable depression/insomnia between MSM-US and NUS. It was hypothesized that (1) MSM-US would have a higher prevalence of probable depression and insomnia than NUS; (2) rumination and catastrophizing would be positively associated with probable depression and insomnia; (3) the differences in probable depression/insomnia between MSM-US and NUS would be partially or fully mediated by both emotional dysregulation strategies (see Fig. 1).

## Materials and methods

### Study design

A cross-sectional study was conducted among male university students in Chengdu and Nanchong cities of Sichuan province, China. Inclusion criteria of MSM-US were (1) aged ≥ 18 years old (2), full-time university students (3), males self-reporting having sex with men in the past six months, and (4) being willing to participate in the present study and provide informed consent. Inclusion criteria of NUS were (1) aged ≥ 18 years old (2), full-time university students (3), males self-reporting having sex with women, but not men, in the past six months, and (4) being willing to participate in the present study and provide informed consent.

### Data collection

This survey was conducted from June to October 2018. The NUS subsample was recruited from three conveniently selected universities. With assistance from schoolteachers and class representatives, one or two classes were randomly selected from various grades and majors as clusters; all students of the selected classes were invited to participate in the present study. The students who were willing to join the survey self-administered a structured questionnaire in the classroom setting. Well-trained field workers briefed the students on the objective, anonymity, and voluntary nature of the study and that they could refuse to participate at any time without negative consequences. No incentives were given to the NUS participants.

For the MSM-US subsample, they were recruited via two sources. First, a community-based survey was conducted with the assistance of a local non-governmental organization (NGO) serving MSM, the Chengdu Tongle Health Counselling Service Center. The NGO staff recruited and trained 20 peer (MSM-US) field workers who recruited the participants via their peer networks and online social networking sites commonly used by local MSM (e.g., WeChat group and Blued); convenience sampling was adopted. Prospective eligible participants contacted the field workers and vice versa. Participants were briefed about the objectives, anonymous and voluntary nature, and logistics of the study. With verbal informed consent, the participants were sent a hyperlink with an online questionnaire that was identical to the above university survey. A WeChat Red Packet of RMB20 (about USD 3) was given to participants as a token of appreciation. Second, students who self-reported having anal sex with men in the past six months in the above university survey were pooled together with the community survey. This study was approved by the Survey Ethics Committee of the Chinese University of Hong Kong (No. SBRE-21-0635).

A total of 2,752 completed questionnaires (305 MSM-US and 2,447 NUS) were collected, of which 221 (8.0%) were removed due to lack of responses in the key variables of insomnia and depression; the remaining 2,531 questionnaires were used in the final data analysis (292 MSM-US and 2,239 NUS).

### Measurements

#### Background information

Information including age, whether being a local student, major subject, year of study, and average monthly expenses was collected.

#### Probable depression

Probable depression was measured by using the 10-item short version of the Center for Epidemiologic Studies Depression Scale (CES-D-10). Its Chinese version demonstrated satisfactory psychometric properties among college students [38]. Sample items included “I could not get going” and “I felt hopeful about the future (reversed)”. The items were rated by using 4-point Likert scales (1 = never to 4 = often); higher scores indicated more depressive symptoms. Probable depression was defined as a CES-D-10 score ≥ 10; this cut-off value has been used in previous studies [39, 40]. The Cronbach’s alpha was 0.783 in this study.

#### Insomnia

Insomnia was measured by using the 7-item Insomnia Severity Index (ISI). Participants were asked to recall sleep difficulties such as sleep onset and maintenance problems in the past two weeks. The Chinese version of the ISI has been validated and showed satisfactory psychometric properties among young adults [41]. A sample item included “Difficulties falling asleep”. The items were rated by using 5-point Likert scales (0 = never to 4 = very serious); higher scores indicated higher levels of insomnia. Cut-off values of ≥ 8, ≥15, and ≥ 22 denoted subthreshold insomnia, clinical moderate insomnia, and clinical severe insomnia, respectively. In this study, the cut-off value of 15 was used, indicating moderate-to-severe clinical insomnia. The Cronbach’s alpha was 0.865 in this study.

#### Emotional dysregulation

Emotional dysregulation was measured by using the two subscales of rumination and catastrophizing of the 18-item Cognitive Emotion Regulation Questionnaire (CERQ) [29], which has been validated among Chinese university students with good psychometric properties [42]. Rumination was assessed by understanding the frequency of past feelings and thoughts recalled. Catastrophizing was measured by the rate of focusing on the negative side of past events. The items were rated by using 5-point Likert scales (1 = almost never to 5 = always); higher scores indicated higher levels of rumination or catastrophizing. The Cronbach’s alpha of the subscales of rumination and catastrophizing were 0.605 and 0.836, respectively.

### Sample size calculation

The sample size planning was conducted by using the module of Tests of Mediation Effect using the Sobel Test in PASS 2021. Assuming power = 0.80, alpha = 0.05, regression coefficients of both independent variable and mediator in indirect effects = 0.20 (small effect size), the proportion of MSM = 0.12, and the standard deviation of mediator = 1, the required sample size was 2,054. The sample size of this study (*n* = 2,531) was hence deemed to be adequate.

### Statistical analysis

Chi-square/t-test was conducted to test the differences in probable depression, insomnia, rumination, and catastrophizing between MSM-US and NUS. Bivariate linear regression analyses were conducted to test the associations between background factors and probable depression/insomnia. Spearman correlation coefficients were generated to test the significance of the interrelationships among the above four key variables. Structural equation modeling (SEM), using the Maximum Likelihood estimator, was conducted to test the mediation effect of the two emotional dysregulation strategies on the differences in probable depression and insomnia between MSM-US and NUS in the overall sample. Three latent variables were created for data analysis. Given that emotional dysregulation is conceptualized as a higher-order construct comprising multiple related yet distinct components [43], a 2-level latent variable of emotional dysregulation based on rumination and catastrophizing was created. The first order latent variable was constructed from the two items of the two original scales; these two first-order variables loaded onto the second-order latent variable of emotional dysregulation. In addition, item parceling in SEM is recommended for scales having over five items. It can minimize the impacts of item-specific measurement errors [44, 45]. Since the scales of depression and insomnia comprise 10 and 7 items, respectively, two latent variables were constructed, each from three randomly grouped item parcels. Satisfactory model fit indices of the SEM included χ^2^/*df* < 5, Comparative Fit Index (CFI) ≥ 0.90, Tucker-Lewis Index (TLI) ≥ 0.90, and Root Mean Square Error of Approximation (RMSEA) ≤ 0.80. The bootstrapping method (*n* = 2,000) was conducted to test the significance of the indirect effects whose 95% CI did not include zero would indicate significant indirect effects. For those background variables having missing responses, the missing responses were grouped into a separate category of that variable; the corresponding recoded dummy variables were hence used for regression and SEM analysis. SEM was conducted by using Mplus 7.0 while other analysis was conducted by using SPSS 26.0. Statistical significance was defined as a two-tailed *p*-value < 0.05.

## Results

### Participants’ characteristics

The mean age (SD; range) of all participants was 20.2 (1.8; 18–30) years; 89.4% were not local students. About one-fifth (20.7%) were first-year students. A majority (91.6%) on average spent ≤ RMB 2000 (about USD 315) per month. About one quarter (24.9%) had subthreshold insomnia while 6% had moderate-to-severe clinical insomnia. The prevalence of probable depression (CES-D-10 ≥ 10) was 37.9% (see Table 1). The MSM-US population was significantly older (mean [SD] = 21.1 [2.8] versus 20.0 [1.6]) and had higher proportions of local students (30.1% versus 7.8%), major subjects of arts (28.8% versus 12.9%), Year-4 or final-year students (25.0% versus 17.5%), and spending less than RMB2,000 per month (27.7% versus 5.4%) than the NUS population (all *p* < 0.001).

**Table 1:** Descriptive statistics

	Overall		MSM-US		NUS		*p*
	*n*/mean	%/SD	*n*/mean	%/SD	*n*/mean	%/SD	
Total	2,531	100.0	292	11.5	2,239	88.5	
Age (years; range = 18–24)	20.2	1.8	21.1	2.8	20.0	1.6	< 0.001
Local students							
Yes	263	10.4	88	30.1	175	7.8	< 0.001
No	2,256	89.1	201	68.8	2,055	91.8	
Missing data	12	0.5	3	1.0	9	0.4	
Major subject							
Arts	373	14.7	84	28.8	289	12.9	< 0.001
Business	194	7.7	39	13.4	155	6.9	
Science	1,842	72.8	131	44.9	1,711	76.4	
Others	87	3.4	22	7.5	65	2.9	
Missing data	35	1.4	16	5.5	19	0.8	
Year of study							
Year 1	524	20.7	34	11.6	490	21.9	< 0.001
Year 2	796	31.5	66	22.6	730	32.6	
Year 3	700	27.7	72	24.7	628	28.0	
Year 4 or final year	464	18.3	73	25.0	391	17.5	
Missing data	47	1.9	47	16.1	0	0.0	
Average monthly expenditure (RMB)							
2,000 or below	2,319	91.6	202	69.2	2,117	94.6	< 0.001
Above 2,000	202	8.0	81	27.7	121	5.4	
Missing data	10	0.4	9	3.1	1	0.0	
Rumination (range = 2–10)	5.7	1.8	6.1	2.0	5.7	1.8	< 0.001
Catastrophizing (range = 2–10)	4.3	1.9	4.7	2.2	4.2	1.9	< 0.001
Insomnia							
Not clinically significant	1,752	69.2	154	52.7	1,598	71.4	< 0.001
Subthreshold	628	24.8	85	29.1	543	24.3	
Moderate	119	4.7	41	14.0	78	3.5	
Severe	32	1.3	12	4.1	20	0.9	
Probable depression*							
Yes	960	37.9	161	55.1	799	35.7	< 0.001
No	1,571	62.1	131	44.9	1,440	64.3	

*Note* SD = Standard deviation; RMB = Renminbi (Chinese Yuan); MSM-US = Men who have sex with men who are university students; NUS = Non-MSM male university students. *Probable depression was defined by the Short Version of the Center for Epidemiologic Studies Depression Scale (CES-D-10) score ≥ 10

### Comparing the levels of key variables between MSM-US and NUS

MSM-US, compared to NUS, showed significantly and substantially higher prevalence of moderate-to-severe clinical insomnia (17.3% versus 4.1%; crude OR = 4.85, 95% CI: 3.38–6.94) and probable depression (55.1% versus 35.7%; crude OR = 2.21, 95% CI: 1.73–2.83) (see Table 1). The MSM-US also showed higher mean scores (SD) for both rumination (6.1 [2.0] vs. 5.7 [1.8], *p* < 0.001) and catastrophizing (4.7 [2.2] vs. 4.2 [1.9], *p* < 0.001) than NM-US (Table 1).

### Associations between background factors and probable depression/insomnia

It is seen from Table 2 that students of older age, non-local, higher grades, and average monthly expenditure > RMB2000 were associated with increased risk of both probable depression and insomnia. The associations involving major subjects were statistically non-significant.

**Table 2:** Associations between background factors and depression/insomnia

	Probable depression				Insomnia			
	B	SE	β	*p*	B	SE	β	*p*
Age (years)	0.18	0.06	0.06	0.002	0.29	0.05	0.11	< 0.001
Local students								
Yes	Ref	-	-	-	Ref	-	-	-
No	1.27	0.34	0.08	< 0.001	0.81	0.31	0.05	0.009
Missing data	1.37	1.50	0.02	0.361	0.42	1.39	0.01	0.760
Major subject								
Arts	Ref	-	-	-	Ref	-	-	-
Business	0.29	0.62	0.02	0.645	0.14	0.57	0.01	0.800
Science	-0.83	0.67	-0.04	0.213	-0.78	0.62	-0.04	0.205
Others	-0.70	0.57	-0.06	0.218	-0.79	0.52	-0.07	0.132
Missing data	1.19	1.04	0.03	0.250	0.40	0.96	0.01	0.679
Year of study								
Year 1	Ref	-	-	-	Ref	-	-	-
Year 2	1.16	0.29	0.10	< 0.001	0.53	0.27	0.06	0.049
Year 3	1.47	0.30	0.13	< 0.001	1.08	0.28	0.10	< 0.001
Year 4 or final year	0.89	0.33	0.07	0.007	0.95	0.30	0.08	0.002
Missing data	5.11	0.78	0.13	< 0.001	3.91	0.72	0.11	< 0.001
Average monthly expenditure (RMB)								
2,000 or below	Ref	-	-	-	Ref	-	-	-
Above 2,000	1.48	0.38	0.08	< 0.001	2.09	0.35	0.12	< 0.001
Missing data	3.11	1.64	0.04	0.058	2.60	1.51	0.03	0.085

*Note* SE = Standard error; Ref = Reference group

### Correlation analysis

As seen from Table 3, the four variables of probable depression, insomnia, rumination, and catastrophizing were positively correlated with each other (*r* ranged from 0.17 to 0.44; all *p* < 0.001).

**Table 3:** Correlation analysis

	Probable depression	Insomnia	Rumination	Catastrophizing
Probable depression	-			
Insomnia	0.44***	-		
Rumination	0.21***	0.17***	-	
Catastrophizing	0.31***	0.19***	0.27***	-

*Note* ***, *p* < 0.001

### SEM

#### Measurement model

Table 4 presents the measurement model of the three latent variables of probable depression, insomnia, and emotional dysregulation, which showed satisfactory model fit indices (χ^2^/*df* = 203.29/30 = 6.77 [*p* < 0.001]; RMSEA = 0.05; CFI = 0.98; TLI = 0.97). The factor loadings ranged from 0.43 to 1.01 with all *p* < 0.001. These results indicated that the latent variables were suitable for conducting SEM.

**Table 4:** Confirmatory factor analysis of the latent variables

Latent variable	Item/package	Factor loading
		β (95% CI)
Probable depression	***Item parcel 1***	0.84 (0.82, 0.87)
	Item “*I was bothered by things that usually don’t bother me*”	
	Item “*I felt depressed*”	
	Item “*I felt hopeful about the future*”	
	Item “*My sleep was restless*”	
	***Item parcel 2***	0.71 (0.68, 0.74)
	Item “*I had trouble keeping my mind on what I was doing*”	
	Item “*I felt that everything I did was an effort*”	
	Item “*I felt fearful*”	
	***Item parcel 3***	0.71 (0.69, 0.74)
	Item “*I was happy (reversed score)*”	
	Item “*I felt lonely*”	
	Item “*I could not ‘get going*’”	
Insomnia	***Item parcel 1***	0.79 (0.76, 0.81)
	Item “*To what extent do you consider your sleep problem to INTERFERE with you daily functioning*”	
	Item “*How NOTICEABLE to others do you think your sleeping problem is in terms of impairing the quality of your life*”	
	Item “*How WORRIED/distressed are you about your current sleep problem*”	
	***Item parcel 2***	0.86 (0.84, 0.88)
	Item “*Difficulty falling asleep*”	
	Item “*How satisfied/dissatisfied are you with your current sleep pattern*”	
	***Item parcel 3***	
	Item “*Difficulty staying asleep*”	0.69 (0.66, 0.72)
	Item “*Problem waking up too early*”	
Emotional dysregulation	***Rumination***	0.56 (0.47, 0.64)
	***Catastrophizing***	0.63 (0.57, 0.70)
Rumination	Item “*I often think about how I feel about what I have experienced*”	0.43 (0.37, 0.49)
	Item “*I am preoccupied with that I think and feel about what I have experienced*”	1.01 (0.90, 1.13)
Catastrophizing	Item “*I keep thinking about how terrible it is what I have experienced*”	0.85 (0.81, 0.89)
	Item “*I continually think how horrible the situation has been*”	0.85 (0.81, 0.89)

*Note* CI = Confidence interval. The model fit indices were: χ^2^/*df* = 203.29/30 = 6.77 [*p* < 0.001]; RMSEA = 0.05; CFI = 0.98; TLI = 0.97

#### Structural model

**Fig. 2:**
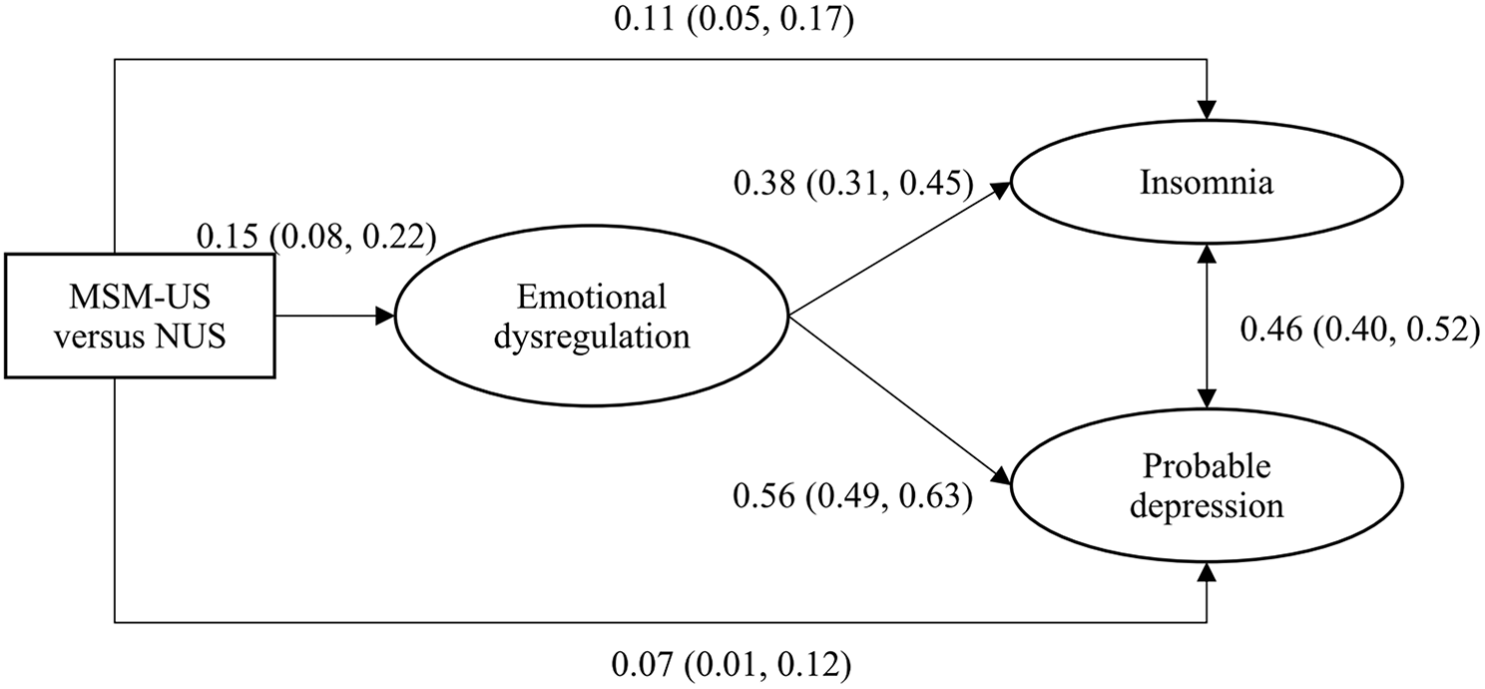
Structural model of the mediation effect (MSM-US = Men who have sex with men who are university students; NUS = Non-MSM male university students. Standardized beta coefficients were reported. The model was adjusted for background factors including age, place of residence, study major, year of study, and average monthly expenses)

Figure 2 presents the SEM results testing the mediation effects of the latent variable of emotional dysregulation on the differences in latent variables of insomnia and probable depression between MSM-US and NUS, adjusting for background factors. The model fit was satisfactory (χ^2^/*df* = 426.54/128 = 3.33 < 5 [*p* < 0.001]; RMSEA = 0.03; CFI = 0.97; TLI = 0.95). MSM-US were more likely than NUS to adopt emotional dysregulation of rumination and catastrophizing approaches (*β* = 0.15; 95% CI: 0.08, 0.22). The latent variable of emotional dysregulation was then positively associated with both insomnia (*β* = 0.38; 95% CI: 0.31, 0.45) and depression (*β* = 0.56; 95% CI: 0.49, 0.63); it also significantly mediated the differences in insomnia (*β* = 0.06, 95% CI: 0.03, 0.09) and probable depression (*β* = 0.08, 95% CI: 0.04, 0.12) between MSM-US and NUS. As the differences in insomnia (*β* = 0.11, 95% CI: 0.05, 0.17) and probable depression (*β* = 0.07, 95% CI: 0.01, 0.12) between MSM-US and NUS were significant, partial mediations were observed; the mediation effect size was 33.6% for insomnia and 55.0% for probable depression.

## Discussion

This study observed high prevalence of probable depression and insomnia in male university students in Sichuan, China and such prevalence was significantly higher in MSM-US than in NUS. In addition, emotional dysregulation of rumination and catastrophizing were positively associated with both probable depression and insomnia and partially explained the differences in the prevalence of probable depression and insomnia between MSM-US and NUS. The findings empirically support Hatzenbuehler’s theoretical framework and provide insights on interventions improving mental problems in male university students, in particular, MSM-US.

In the overall sample including MSM-US and NUS, the high prevalence of probable depression (about 40%) was observed, which corroborated previous studies of general Chinese university students (42.8%) [39], but was much higher than that of university students in the U.S. (17.8%) [46] and Malaysia (29.4%) [47]; these studies used the same cut-off point of CES-D-10 ≥ 10. In contrast, based on ISI ≥ 15, the prevalence of moderate-to-severe insomnia (6%) in this study was slightly lower than that of college students in Lebanon (10.6%) [48], India (9.1%) [49], and the U.S. (22.5%) [50]. The alarmingly high prevalence of depression and relatively low prevalence of insomnia in Chinese university students might be associated with high academic stress and the living conditions on campus. Due to the highly competitive educational environment in China, university students may face multifaceted academic stressors, increasing the risk of depression and insomnia [51]. Nonetheless, most Chinese university students live in dormitories having four to six persons per room, resulting in less personal control over their sleep environment and then a more structured and disciplined sleep pattern (e.g., fixed sleep time due to fixed time for turning on and off the dormitory lights). Such may mitigate the impacts of academic stress on sleep to some extent, and this speculation needs confirmation in future studies. Male university students in Sichuan, China who were older, non-local students, or had a moderate level of monthly expenditure warrant more attention in mental health interventions, as such background factors were significantly associated with probable depression and insomnia in this study.

Supporting our hypothesis and the Sexual Minority Theory [1], MSM-US had a much higher prevalence of probable depression and insomnia than NUS, which corroborates the findings of previous comparative studies [24–26]. Besides common stressors of university students, MSM-US faced other stressors specific to their MSM status that would increase their risk of mental problems [1]. For instance, although it is clear in China that same-sex behavior is not an illness, disclosure of one’s MSM status is still a big challenge due to potential societal and internal stigma [52]. The process of coming out could be a significant source of stress and generate fear of rejection or negative repercussions, while those who hide their identity could experience chronic stress and anxiety [53, 54]; both scenarios predicted mental problems including depression and insomnia [1].

In addition, a novel finding of this study confirmed the above postulation by revealing that rumination and catastrophizing significantly and partially mediated the differences in probable depression/insomnia between MSM-US and NUS. First, the findings indicate that MSM-US was more likely to adopt rumination and catastrophizing approaches, which supports Hatzenbuehler’s psychological mediation framework postulating that multiple minority stress (e.g., discrimination) would lead to negative affect and difficulties in emotional regulation among MSM [30]. Second, emotional dysregulation strategies were significantly and positively associated with both probable depression and insomnia, confirming the common understanding that emotional dysregulation was a risk factor of mental problems [33, 35, 36]. Notably, the effect sizes of both mediation paths were substantial, suggesting that modification of emotional dysregulation approaches may greatly reduce mental problems among MSM-US. However, partial instead of full mediations imply the existence of other unstudied potential mediators, such as interpersonal factors of social support and loneliness, which were associated with both MSM status and mental outcomes.

Based on the findings of this study, mental health interventions targeting MSM-US and NUS are recommended to address common stressors for university students, MSM-specific stressors, and emotional dysregulation of rumination and catastrophizing, taking into account the Chinese education context. First, health education is greatly needed for skills training in recognizing and addressing stressful life events in and out of campus (e.g., academic competition, social conflicts, and life transition) [55–57] to tackle the common stressors. Second, as for MSM-specific stressors, concerted efforts among stakeholders (e.g., individuals, families, government, civil societies, and communities) are required to change public attitudes toward sexual minority groups and coordinate strategic communication to reduce social discrimination and internalized stigma. University-based interventions are also needed to establish a more accepting environment for sexual minorities. Third, a review summarized a series of effective interventions reducing emotional dysregulation, including rumination-focused cognitive-behavioral therapy (CBT) that used novel constructs of functional analysis and contextual approaches, metacognitive therapy, mindfulness-based CBT, and cognitive control training [58]. Other potentially effective interventions include forming and changing adaptive coping strategies (e.g., acceptance and positive reframing), positive self-affirmation, and good self-care practices. Such may be integrated into the existing mental health service in the universities.

The present study has novelty but several limitations. First, temporal or causal relationships cannot be established due to the cross-sectional nature of this study; longitudinal studies are warranted to verify the findings. Second, social desirability bias may exist. For instance, individuals with depressive symptoms might have internalized stigma and thus be less likely to report these symptoms. Third, since no sampling frame exists for MSM which is a hard-to-reach population, like most of other MSM studies [59], convenience sampling for the MSM population was used in this present study. We used dual university-based and community-based approaches with consistent inclusion criteria, survey instruments, and data management protocols to recruit MSM-US to increase the inclusiveness. Notably, a small incentive of about USD 3 was given to the participants of the community-based group as a token of appreciation, but not to those of the university-based group. We believe that the amount given was nominal and would not play a major role in the decision-making of whether to join the study. The university-based survey involved selecting classes as clusters, which may introduce correlations among responses within these clusters. However, as the cluster information was not collected, specific statistical methods were not employed to reduce this potential non-independence of observations. Such may affect the precision of our estimates. In addition, the studied population was conveniently selected from three universities in one Chinese province, and the sample size of MSM-US was much smaller than that of NUS. These potential selection bias and sample imbalance further indicate caution in generalizing the results to other regions and populations. Fourth, the prevalence of probable depression and moderate-to-severe clinical insomnia might be overestimated as classifications were based on screening tools instead of clinical diagnosis, although both CES-D-10 and ISI were commonly used [39, 47, 49, 50]. Last, other potential confounders (e.g., emotional state and recent sexual behaviors) and mediators (e.g., social support and other emotional dysregulation strategies such as self-blame) were not investigated in this study and should be taken into account in future relevant studies.

## Conclusions

In conclusion, this study was one of the very few studies that directly compared the prevalence of probable depression and insomnia between MSM-US and NUS in Sichuan, China and found a much higher prevalence of probable depression and insomnia among MSM-US than NUS. Supporting Hatzenbuehler’s psychological mediation framework and emotion-based theories of depression, increased levels of emotional dysregulation strategies of rumination and catastrophizing explained a sizable proportion of the differences in probable depression and insomnia between MSM-US and NUS. Future longitudinal and intervention studies are warranted to verify these findings and explore other potential mediators in both MSM and other sexual minority groups.

## Data Availability

The data would be available upon reasonable request to the corresponding author.

## Code availability

The analysis code is available upon reasonable request.

## Abbreviations

MSM-US: Men who have sex with men who are university students
SEM: Structural equation modeling
MSM: Men who have sex with men
NUS: Non-MSM male university students
NGO: Non-governmental organization
CES-D-10: The 10-item Short version of the Center for Epidemiologic Studies Depression Scale
ISI: Insomnia Severity Index
CERQ: Cognitive Emotion Regulation Questionnaire
CFI: Comparative Fit Index
TLI: Tucker-Lewis Index
RMSEA: Root Mean Square Error of Approximation
CI: Confidence interval
SD: Standard deviation
OR: Odds ratio
CBT: Cognitive Behavioral Therapy
